# Second generation *Pichia pastoris* strain and bioprocess designs

**DOI:** 10.1186/s13068-022-02234-7

**Published:** 2022-12-29

**Authors:** Burcu Gündüz Ergün, Kübra Laçın, Buse Çaloğlu, Barış Binay

**Affiliations:** 1 National Nanotechnology Research Center (UNAM), Bilkent University, 06800 Ankara, Turkey; 2 Biotechnology Research Center, Ministry of Agriculture and Forestry, 06330 Ankara, Turkey; 3 Department of Bioengineering, Gebze Technical University, 41400 Gebze, Kocaeli Turkey; 4 BAUZYME Biotechnology Co., Gebze Technical University Technopark, 41400 Gebze Kocaeli, Turkey

**Keywords:** *Pichia pastoris* (*Komagataella phaffii*), Heterologous protein production, Agri-food wastes and by-products, Industrial wastes and by-products, Low-cost substrates, Lignocellulosic biomass, Crude glycerol, Circular bioeconomy

## Abstract

Yeast was the first microorganism used by mankind for biotransformation processes that laid the foundations of industrial biotechnology. In the last decade, *Pichia pastoris* has become the leading eukaryotic host organism for bioproduct generation. Most of the *P. pastoris* bioprocess operations has been relying on toxic methanol and glucose feed. In the actual bioeconomy era, for sustainable value-added bioproduct generation, non-conventional yeast *P. pastoris* bioprocess operations should be extended to low-cost and renewable substrates for large volume bio-based commodity productions. In this review, we evaluated the potential of *P. pastoris* for the establishment of circular bioeconomy due to its potential to generate industrially relevant bioproducts from renewable sources and waste streams in a cost-effective and environmentally friendly manner. Furthermore, we discussed challenges with the second generation *P. pastoris* platforms and propose novel insights for future perspectives. In this regard, potential of low cost substrate candidates, i.e., lignocellulosic biomass components, cereal by-products, sugar industry by-products molasses and sugarcane bagasse, high fructose syrup by-products, biodiesel industry by-product crude glycerol, kitchen waste and other agri-food industry by products were evaluated for *P. pastoris* cell growth promoting effects and recombinant protein production. Further metabolic pathway engineering of *P. pastoris* to construct renewable and low cost substrate utilization pathways was discussed. Although, second generation *P. pastoris* bioprocess operations for valorisation of wastes and by-products still in its infancy, rapidly emerging synthetic biology tools and metabolic engineering of *P. pastoris* will pave the way for more sustainable environment and bioeconomy. From environmental point of view, second generation bioprocess development is also important for waste recycling otherwise disposal of carbon-rich effluents creates environmental concerns. *P. pastoris* high tolerance to toxic contaminants found in lignocellulosic biomass hydrolysate and industrial waste effluent crude glycerol provides the yeast with advantages to extend its applications toward second generation *P. pastoris* strain design and bioprocess engineering, in the years to come.

**Graphical Abstract:**

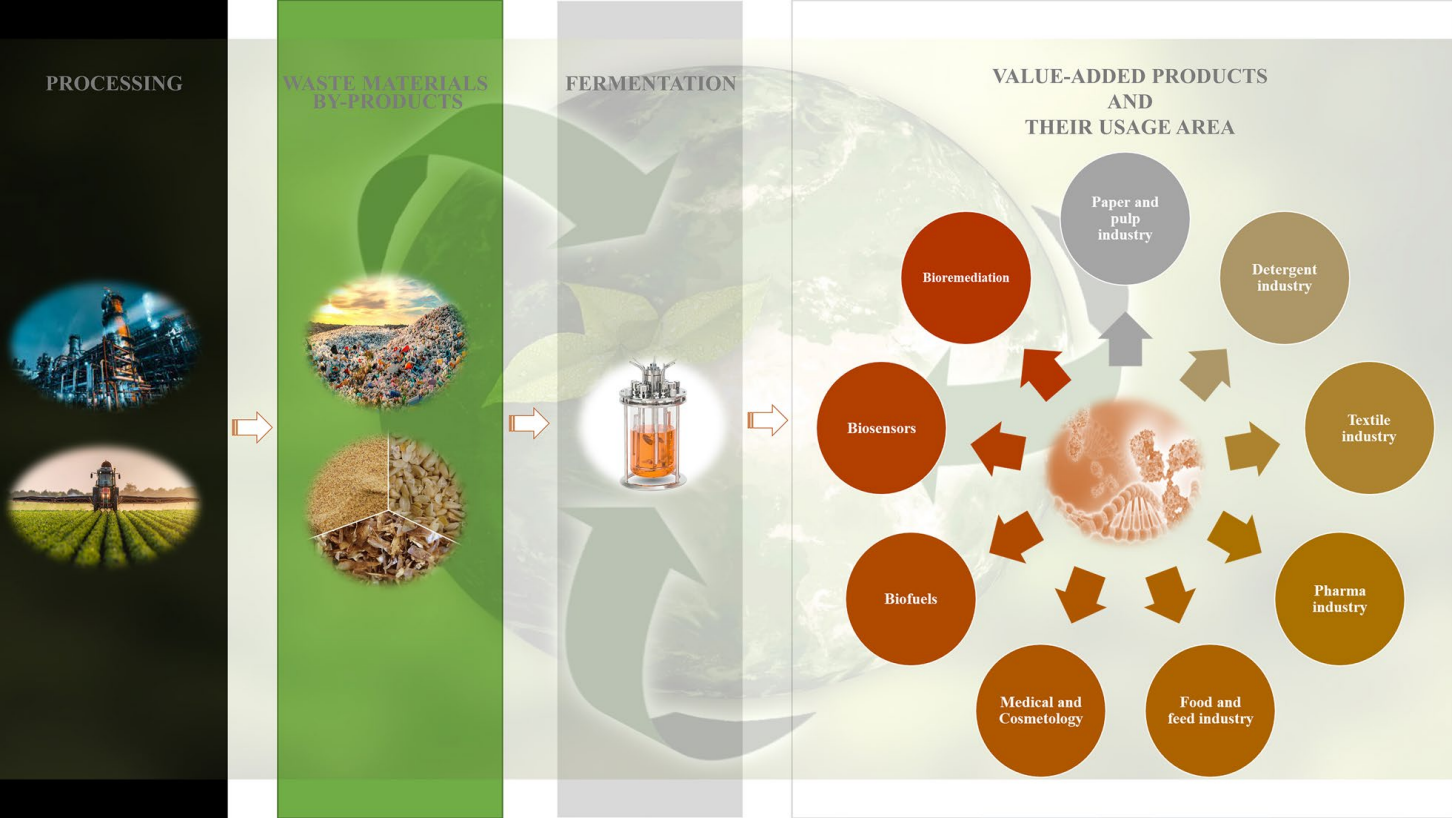

## Introduction

Non-conventional methylotrophic yeast *Pichia pastoris* (syn *Komagataella phaffii*) was first isolated from chestnut tree exudate in France in 1919. Around mid-twentieth century, after the discovery of its methanol utilizing ability [1] Philips Petroleum Company employed it to produce single cell protein (SCP) as an animal feed additive; however, due to increased methanol prices resulting from oil crisis in 1973, SCP process became uneconomical. *P. pastoris* was transformed into a recombinant protein expression platform in the 1980s [2]. Since then, more than 5000 recombinant proteins have been expressed, and a number of *P. pastoris* bioproducts have been approved by regulatory agencies, such as European Medicine Agency (EMA), the European Food Safety Authority (EFSA), and USA Food and Drug Association (FDA). In recombinant protein production, the number of research outputs with *P. pastoris* has outperformed conventional yeast *Saccharomyces cerevisiae* and makes *P. pastoris* the most commonly used eukaryotic host system [3]. Currently, around 70 different biomolecules including recently approved monoclonal antibody eptinezumab and nanobody caplacizumab Cablivi are on the market or in late stage development. Furthermore, *P. pastoris* has qualified presumption of safety (QPS) status by EFSA for production of food and feed enzymes and it can be applied for food and feed products based on microbial biomass [4].

**Fig. 1:**
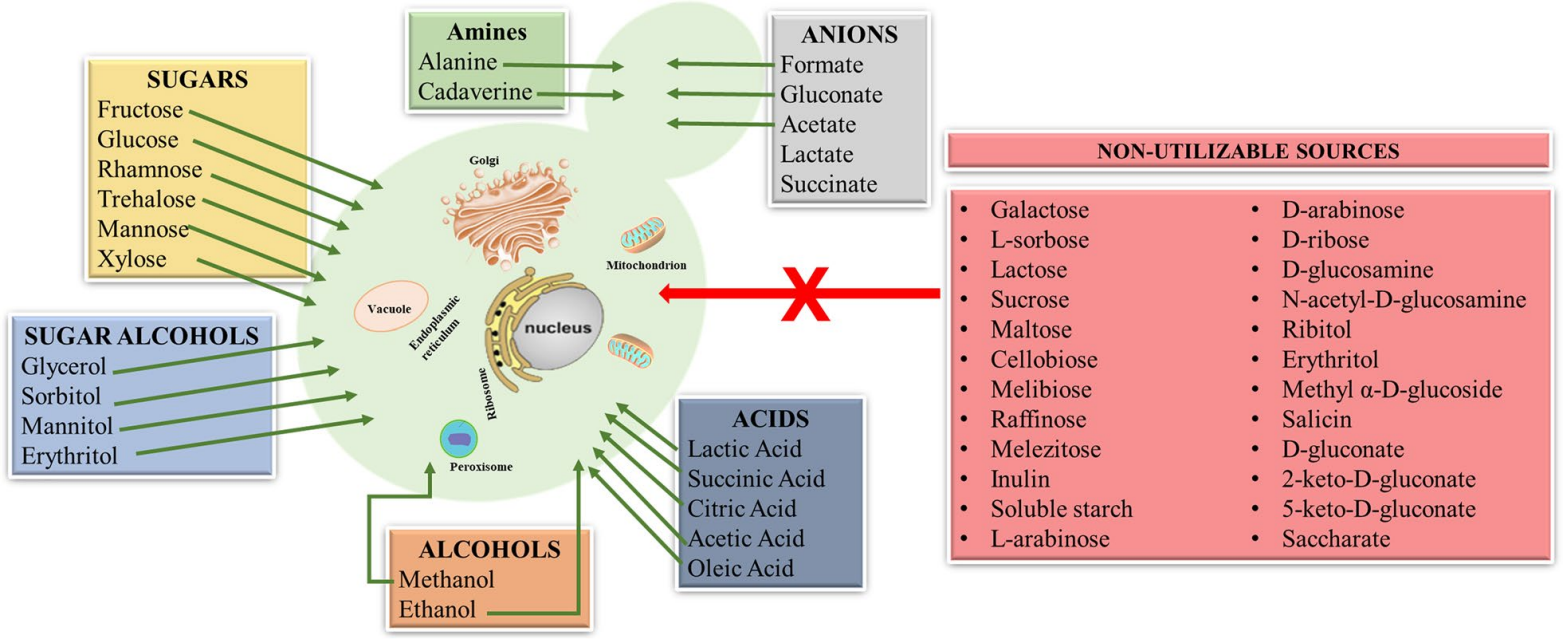
Different carbon sources for *P. pastoris* cultivation. Green arrow indicates utilizable carbon sources, while red arrow indicates non-utilizable carbon sources

*P. pastoris* is an attractive chassis for the production of both high-value (e.g., antibodies, hormones, vaccines) and low-value (e.g., food and feed enzymes, processing aids) bioproducts. *P. pastoris* bioprocess operations mostly employ strong methanol-inducible alcohol oxidase 1 promoter (P_*AOX1*_) and constitutive glyceraldehyde 3-phosphate dehydrogenase promoter (P_*GAP*_). New promoters also have been engineered for different regulation mechanisms and enhanced strength to extend the *P. pastoris* bioprocess operation widows [5–8]. Methanol, glucose and glycerol are the most commonly used carbon sources in *P. pastoris* bioprocess operations,. Meanwhile, *P. pastoris* can utilize other hexose and pentose sugars, i.e., fructose [9, 10], rhamnose [11, 12], mannose [13], trehalose [11, 14] and also various sugar alcohols, such as D-mannitol [11, 15] and sorbitol [16]. Consumption of ethanol [6], lactate, succinate [11], lactic acid, acetic acid [17], formate [16, 18], succinic acid, citric acid [19], gluconate [20], alanine [15], oleic acid [21, 22], and acetate [15, 17, 23] are also proved. On the other hand, *P. pastoris* cannot utilize galactose, L-sorbose, lactose, sucrose, maltose, cellobiose, melibiose, raffinose, melezitose, inulin, soluble starch, L-arabinose, D-arabinose, D-ribose, D-glucosamine, N-acetyl-D-glucosamine, ribitol, methyl α-D-glucoside, salicin, D-gluconate, 2-keto-D-gluconate, 5-keto-D-gluconate, saccharate [11]. Utilizable and non-utilizable carbon sources of *P. pastoris* are schematically represented in Fig. 1.

Around the world, there is a growing interest in the circular bioeconomy due to the current unsustainable model of production and consumption based on increased use and depletion of resources [24]. Climate change and ever-growing population create high demand for commodity products and deplete sources [25]. To establish a robust bioeconomy, economically viable conversion of low-cost renewable feedstock into bioproducts and biofuels is of utmost importance. In this regard, non-conventional yeasts are attracting more and more attention due to their potential to metabolize complex carbon molecules, alternative metabolic routes for new product formation, and their ability to cope with industrial process conditions. Second generation *P. pastoris* platforms define sustainable bioprocess operations with renewable sources or waste streams to generate biomass and tailor-made bioproducts.

Circular economy model indicates recycling of the by-products and wastes of the different industrial manufacturing processes as outcome of cost management strategies contributes to eco-friendly and sustainable biotechnological production [26]. Different waste streams are formed as a result of industrial, agri-food operations, and modern lifestyle [26–30]. Agri-food wastes originate throughout the whole food-supply chain, from production to post-harvesting, industrial processing, distribution, domestic processing, and consumption, with wastage volumes differing among phases and food commodities [31]. Each commodity group creates specific by-products. Fruit and vegetable processing generates large amount of pomace, seeds, peelings, trimmings, stones, leaves and stems, while cereal grain milling produces germ, bran, husks, hulls, broken grain and powders. Crude glycerol is the major by-product of biodiesel industry that is formed during methanolysis of vegetable oils [32, 33]. Fermentation, composting, and usage as animal feed are some of the utilization methods of by-products and waste materials [34]. Million metric tons of wheat straw, rice straw, molasses, and other agri-food wastes and by-products have been emerging annually (Table 1). In addition, the global wasted quantity of household food and beverage is approximately 1.3 billion tonnes each year [35]. Furthermore, these wastes and by-products are rich in carbon, and their removal is a big challenge. Disposal of untreated agricultural by-products/wastes and their burning or dumping processes lead to environmental pollution due to releasing high carbon into the air thus causing climate change and leads health problems [28, 36].

**Table 1:** Global annual production and composition of agri-food wastes and by-products

Agri-food wastes and by-products	Composition	Global annual production amount	References
Corn cob	Cellulose, 40–45% Hemicellulose, 31–39% Lignin, 14–18%	200 million tons	[52, 55]
Corn bran	Hemicellulose, 700 g/kg Cellulose, 280 g/kg	3 million tons (dry mass)	[56]
Wheat bran	Starch, 14–25% Non-starch carbohydrates, 5–60% Fat, 3–4% Protein, 13–18% Mineral, 3–8%	150 million tons	[57, 58]
Rice bran	Carbohydrate, 34–62% Protein, 11–15% Lipids, 15–20% Crude fiber, 7–11% Ash, 7–10%	29.3 million tons	[59, 60]
Bean pulp	Protein, 40–48% Amino acid, 3.6–4.4%	–	[61]
Maize meal	Carbohydrate, 90.9% Protein, 7.1% Fat, 1.52%	66,332.9 tons	[62, 63]
Miscellaneous waste carbohydrates	Glucose, Fructose, Oligosaccharides	415,000 tons	[64]
Napier grass	Cellulose, 35.0–39.4% Xylan, 19.2–23.4% Lignin, 15.3–19.3%	–	[65]
Sugarcane bagasse	Cellulose, 50% Hemicellulose, 25% Lignin, 25%	6 million tons	[66, 67]
Sugar beet molasses	Total sugars (sucrose, glucose, fructose), 50% Suspended colloids, Heavy metals, Vitamins, Nitrogenous compounds	500,000 tons^a^	[68]

^a^Data show the annual production amount of Colombia

Agri-food waste materials generally comprise 25–30% hemicellulose, 40–50% cellulose, 15–20% lignin, ash and moisture [28, 37]. A variety of microorganisms including fungi, bacteria, and yeast species catalyse conversion of low-cost substrates into value-added products [38–42]. Alternative to commercial fermentation media components, industrial by-products and agri-food wastes could be used as carbon, nitrogen, or other essential nutrient sources in the development of second generation bioprocess operations.

This review article focuses on the second generation *P. pastoris* platforms which have a great potential to generate industrially relevant compounds from renewable sources and wastes/by-products in a cost-effective and environmentally friendly manner. Until now, research has conducted on *P. pastoris* cell growth and bioproduct generation using low-cost substrates are critically evaluated in the article (compiled in Table 2).

**Table 2:** *Pichia pastoris* cell growth and bioproduct generation with a variety of wastes and by-products

Source	Strain	Promoter	Product	Bioreactor	Bioprocess operation mode	Yield	Productivity	References
Agri-food industry by-products and wastes								
Wood sugar xylose								
Corncob hydrolysate	GS115 and evolved strain (GS30)	–	β-Galactosidase β-Mannanase	Shake flask	Fed-batch process	72.6 U/mL 45.1 U/mL	–	[52]
Xylose	PichiaPink	P_*MOX*_	Endoglucanase 3 EndoglucanaseII	Shake flask	-	No expression	–	[54]
Xylose	GS115	P_*GAP*_	β-Mannanase	Shake flask	Batch process	> 60 U/mL	–	[104]
Cereal by-products								
Pakchong 1	–	–	Xylanases	Shake flask	SoSF	31.44 ± 0.88 U/g	0.2620 ± 0.02 U/g.h^−1^	[80]
Sugarcane leaves	–	–	Xylanases	Shake flask	SoSF	29.72 ± 0.35 U/g	0.2477 ± 0.01 U/g.h^−1^	[80]
Merkeron	–	–	Xylanases	Shake flask	SoSF	34.10 ± 0.88 U/g	0.2842 ± 0.01 U/g.h^−1^	[80]
Alafal	–	–	Xylanases	Shake flask	SoSF	26.84 ± 0.92 U/g	0.2237 ± 0.09 U/g.h^−1^	[80]
Alafal	–	–	Xylanases	Shake flask	SmF	56.08 ± 0.34 U/g	0.4674 ± 0.03 U/g.h^−1^	[80]
Merkeron	–	–	Xylanases	Shake flask	SmF	49.37 ± 0.72 U/g	0.4114 ± 0.05 U/g.h^−1^	[80]
Pakchong 1	–	–	Xylanases	Shake flask	SmF	49.48 ± 0.30 U/g	0.4124 ± 0.05 U/g.h^−1^	[80]
Sugarcane leave	–	–	Xylanases	Shake flask	SmF	61.21 ± 0.52 U/g	0.5101 ± 0.03 U/g.h^−1^	[80]
Merkeron	–	–	Cellulase	Shake flask	SoSF	11.85 ± 0.35 U/g	0.0988 ± 0.01 U/g.h^−1^	[80]
Sugarcane leaves	–	–	Cellulase	Shake flask	SoSF	10.37 ± 0.49 U/g	0.0864 ± 0.05 U/g.h^−1^	[80]
Alafal	–	–	Cellulase	Shake flask	SoSF	8.89 ± 0.61 U/g	0.0618 ± 0.03 U/g.h^−1^	[80]
Pakchong 1	–	–	Cellulase	Shake flask	SoSF	9.44 ± 0.37 U/g	0.0787 ± 0.02 U/g.h^−1^	[80]
Merkeron	–	–	Cellulase	Shake flask	SmF	39.21 ± 0.45 U/g	0.3268 ± 0.03 U/g.h^−1^	[80]
Sugarcane leaves	–	–	Cellulase	Shake flask	SmF	39.72 ± 0.30 U/g	0.3310 ± 0.02 U/g.h^−1^	[80]
Alafal	–	–	Cellulase	Shake flask	SmF	29.51 ± 0.65 U/g	0.2459 ± 0.01 U/g.h^−1^	[80]
Pakchong 1	–	–	Cellulase	Shake flask	SmF	31.37 ± 1.16 U/g	0.2614 ± 0.02 U/g.h^−1^	[80]
Crude glycerol, Soybean hydrolysate and Rice bran	KM71	P_*AOX1*_	Xylanase	Shake flask	Methanol fed-batch process	1383.9 U/mL	–	[82]
Wheat bran hydrolysate	–	P_*AOX1*_	Xylanase B	Shake flask	Methanol fed-batch	1059.8 U/mL	–	[103]
Maize meal bean pulp	GS115	P_*AOX1*_	Xylanase	Shake flask	Methanol fed-batch process	3273.0 U/mL	–	[81]
Wheat bran	–	P_*AOX1*_	Xylanase	Bioreactor	Methanol fed-batch process	3,683 mU/mL	–	[79]
Wheat bran	X33	P_*AOX1*_	Xylanase	Bioreactor	Methanol fed-batch process	560.7 U/mL	–	[78]
Corncob	X33	P_*AOX1*_	Xylanase	Shake flask	Methanol fed-batch process	Decreased by the rate of 70.31%	–	[78]
Cottonseed hull	X33	P_*AOX1*_	Xylanase	Shake flask	Methanol fed-batch process	Positive effect	–	[78]
Corn bran	X33	P_*AOX1*_	Xylanase	Shake flask	Methanol fed-batch process	Positive effect	–	[78]
Corn processing wastewater	GS115	P_*AOX1*_	Lipase	Shake flask	Methanol fed-batch process	163 U/L	–	[77]
Sugar industry by-products								
Miscellaneous waste carbohydrates of high fructose syrup	–	P_*GAP*_ P_*AOX1*_	Endo-β-1,3-glucanase	Bioreactor	Fed-batch process	171.8 U/ml 212.3 U/mL	–	[105]
Sugarcane bagasse	X33	P_*GAP*_	Xylonic acid	Shake flask	Batch process	0.43 ± 0.06 g/g	0.16 ± 0.02 g/L.h	[106]
Sugar beet molasses	–	P_*GAP*_	Recombinant human growth hormone	Bioreactor	Fed-batch process	625 mg/dm^3^	–	[107]
Molasses	GS115	P_*FLD1*_	α-Amylase	Bioreactor	Methanol Fed-batch process	120 mg/L	–	[108]
Biodiesel industry by-products and wastes								
Crude glycerol	BG-10, BG-11, BG-16	P_*D915*_	Laccase	Shake flask	Batch process	2343 ± 60 U/L	–	[109]
Crude glycerol	SMD1168H	P_*GAP*_	Lectin	Bioreactor	Glycerol fed-batch process	265 ± 13 mg/L	2.04 mg/L.h	[110]
Crude glycerol	GS115	P_*AOX1*_	Lipase	Shake flask	Methanol fed-batch process	1437 U/mL (15,977 U/mg)	–	[111]
Sago bioethanol liquid waste	GS115	P_*AOX1*_	Laccase	Shake flask	Methanol fed-batch process	0.00159 U/ mL	–	[112]
Crude glycerol	X33	P_*AOX1*_	β-Mannanase	Bioreactor	Methanol fed-batch process	10,900 U/mL	–	[113]
Crude glycerol	X33	P_*PGK1*_	Lipase	Bioreactor	Glycerol fed-batch process	50,041 U/L	–	[114]
Waste glycerol	X33	–	–	Bioreactor	Batch process	Positive effect on growth	–	[115]
Glycerin byproduct	X33	P_*PGK*_	α-Amylase	Bioreactor	Batch process	~ 3.5 U/mL	–	[116]
Waste glycerol	GS115	–	–	Shake flask	Batch process	Positive effect on growth	–	[117]
Co-culture studies for valorisation of wastes and by-products								
Kitchen waste	GS115 co-culture with *Bacillus amyloliquefaciens*	P_*AOX1*_	Amylase	Shake flask	Methanol fed-batch process	385.4 U/mL	–	[118]
Kitchen waste	GS115 co-culture with *Bacillus amyloliquefaciens*	P_*AOX1*_	Glucosidase	Shake flask	Methanol fed-batch process	247.3 U/mL	–	[118]
Kitchen waste	GS115 co-culture with *Bacillus amyloliquefaciens*	P_*AOX1*_	Lipase	Shake flask	Methanol fed-batch process	90 U/mL	–	[118]
Xylose	GS115 co-culture with *S. cerevisiae*	P_*AOX1*_	β-Xylosidase	Shake flask	Methanol fed-batch process	3.02 ± 0.030 U/mL	–	[37]
Xylose	GS115 co-culture with *S. cerevisiae*	P_*AOX1*_	Endo-1,4-β-d-xylanase	Shake flask	Methanol fed-batch process	1.52 ± 0.035 U/mL	–	[37]
Wheat straw	GS115 co-culture with *S. cerevisiae*	P_*AOX1*_	Ethanol	–	Methanol fed-batch process	32.6 g/L	–	[37]
Metabolic engineering studies for low-cost substrate utilization								
Sugarcane trash hydrolysates	KM71	P_*GAP*_	Isobutanol	Shake-flask	Batch process	48.2 ± 1.7 mg/L	–	[119]
CO_2_	CBS7435	P_*AOX1*_, P_*DAS1*_ P_*DAS2*_	Growth	Bioreactor	Fed-batch process	µ was increased from from 0.008/h to 0.018/h	–	[120]
Crude hemicellulose hydrolysate	GS115	P_*AOX1*_	Formate dehydrogenase Xylose reductase Glucose dehydrogenase	Bioreactor	Methanol fed-batch process	0.34 ± 0.02 U/mg 62.1 ± 7.1 U/mg 17.4 ± 1.0	–	[121]
Acetate	GS115	P_*GAP*_	6-Methylsalicylic acid	Shake flask	Batch process	Enhanced	–	[122]
Cellobiose -carboxymethyl cellulose	BG10	P_*AOX1*_	Endoglucanase, β-glucosidase Exoglucanase	Shake flask	Batch process	Positive effect	–	[123]
Xylan, carboxymethyl cellulose	X33	P_*ADH2*_	Chimeric endoglucanase Xylanase	Shake flask	Batch process	1.4 U/mg 1.8 U/mg	–	[124]

*SoSF* solid-state fermentation, *SmF* submerged fermentation

### Valorisation of renewable raw materials, wastes and by-products with *P. pastoris*

#### Wood sugar xylose

Lignocellulose is the most abundant biomass on Earth with an annual global production of about 181.5 billion tonnes [43]. Of these, about 7 billion tonnes from agricultural, grass or forest land are currently used as fodder or for energetic and material purposes. In addition, about 4.6 billion tonnes of lignocellulosic biomass residues are produced as agricultural residues, of which only about 25% are used intensively [44]. Lignocellulosic biomass is composed of cellulose which is embedded in complex hemicellulose and lignin matrices, and consists of ca. 40% cellulose, 33% hemicellulose, and 23% lignin by dry weight [45]. Xylan, a heterogeneous polysaccharide composed of β-1,4-linked xylose (C_5_H_10_O_5_) backbone and commonly found in agricultural wastes, is the major component of hemicellulose and accounts for one-third of all renewable organic carbon on earth. Xylose is the most abundant pentose sugar in hemicellulose and second only to glucose in natural abundance [46]. Bacteria, filamentous fungi and some yeast species could utilize xylose [37, 47–49]. Xylose is a promising renewable carbon source for bioproduct generation. *Scheffersomyces, Meyerozyma, Candida, Spathaspora,* and *Kluyveromyces* are xylose-fermenting yeast genera, while *Komagataella, Yarrowia,* and *Ogataea* are either xylose utilization deficient or have very low utilization rates [50].

*P. pastoris* GS115 can utilize xylose with a very low specific growth rate (0.0075 h^−1^, approximate doubling time 92 h) [51]. The Adaptive Laboratory Evolution was carried out to strengthen the xylose utilization efficiency of *P. pastoris* GS115 and three different evolved strains GS30 (mutant of 30th generation), GS50 (mutant of 50th generation), and GS70 (mutant of 70th generation) were generated [52]. All the evolved strains showed improved xylose utilization compared to wild-type strain, while the GS50 strain was optimal in terms of xylose utilization and biomass yield. The performance of evolved strains was analysed on corncob hydrolysate, since it contains additional carbon sources to xylose including glucose, arabinose, rhamnose, mannose and galactose. GS30 showed the highest biomass yield (similar to wild-type strain) on corncob hydrolysate possibly due to more efficient utilization of other carbon sources, whereas GS50 and GS70, respectively, showed 21–33% decline in biomass accumulation compared to the wild-type strain. GS30 strain was further engineered to produce recombinant β-galactosidase and β-mannanase under *AOX1* promoter control. GS30 enzyme secretion improved 35–53% on Buffered Complex Xylose Medium and corncob hydrolysate compared to wild-type cell [52].

Three different *Komagataella* species, i.e.,* K. phaffii* X-33, *K. pastoris* CBS 704 and *K. populi* CBS 12362, were investigated for xylose assimilation [53]. *K. phaffii* X-33 was the best xylose consumer using up an average 95.70% ± 3.2 of the available xylose for nearly one doubling within 10 days of cultivation, while *K. pastoris* CBS 704 had the slowest growth and xylose uptake in comparison the other two strains. *K. populi* CBS 12362 showed the fastest growth, consuming 61.4% ± 3.0 of the available xylose in the fermentation media. Xylitol production was identified with all three strains indicating xylose assimilation through an oxidoreductase pathway [53].

Metabolic engineering approaches were also applied to design efficient xylose utilizing *P. pastoris* strains. Xylose utilization pathway was introduced into *P. pastoris* GS115 by overexpressing the anaerobic rumen fungus *Orpinomyces* spp. xylose isomerase and/or endogenous xylulokinase gene under *GAP* promoter besides evolutionary engineering strategies [51]. A significant difference in xylose utilization was not determined in overexpression of xylose isomerase, xylulokinase and a combination of these two enzymes. Evolutionary engineering remarkably increased specific growth rates of wild-type, xylulokinase overexpressing and xylose isomerase overexpressing *P. pastoris* strains by 49%, 92% and 80%, respectively. Although cell growth was not improved in the simultaneous xylulokinase and xylose isomerase overexpression strain after 50 generations of evolution, xylose utilization rate increased by 56% [51]. With these metabolic engineering applications, nearly twofold higher cell yield and significantly enhanced recombinant protein production on xylose media were achieved.

Seeking novel regulated promoters in *P. pastoris* led Mombeni et al. [54] to evaluate the *Hansenula polymorpha* methanol oxidase (*MOX*) promoter and interestingly they found that in *P. pastoris* P_*MOX*_ is repressed by xylose and sorbitol.

### Cereal by-products

Cereals were the first crops to be cultivated by mankind and constitute the majority of global staple foods, e.g., corn, rice and wheat. Cereals belong to the grass family, and have edible seeds with high carbohydrate content. Cereal processing produces large volumes of by-products including corn bran, wheat bran, and corncob [69]. There has been an increasing interest in valorisation of cereal by-products [70–72]. Bran, the outer layer of cereals, is usually discarded during milling process. Corn endosperm is used as main component of grit and corn oil production, while corn bran and corn flour are removed as by-products [73]. Corn bran is produced in yields of 60–70 g/kg, with a total production of 3 × 10^6^ dry tonnes per year [74]. Hemicellulose and arabinoxylan are fundamentally obtained from corn bran and their hydrolysates provide xylose, arabinose, glucose, galactose, rhamnose and mannose. However, during acid or base pre-treatment stages inhibitory compounds formation, such as furfural creates negative effect on microbial growth and production [74, 75]. Another agro-waste corncob is produced more than 200 million tons annually (Elegbede, Ajayi and Lateef 2021). 220 g corncob with 69.2% cellulosic content is obtained from 1 kg corn [76]. Similar to corn bran, 150 million tons of wheat bran composed of 14–25% starch, 5–60% non-starch carbohydrates, 3–4% fat, 13–18% protein and 3–8% minerals are generated annually [58]. Corn bran and wheat bran can be used for animal feeding [57].

Yan et al. [77] employed corn processing wastewater with various commercial nitrogen and salt supplements as a medium for the production of recombinant *Geotrichum* sp. lipase by *P. pastoris.* Supplement concentrations with corn processing wastewater were optimized by Plackett–Burman design and Response Surface Methodology. 163 U.L^−1^ lipase activity was achieved throughout the optimization process that enhanced the total lipase production by 4.94-fold that reached higher yield compared to the conventional Buffered Complex Methanol Medium and high salt medium.

Carbon sources offer both energy and carbon skeletons for growth of *P. pastoris* cells and expression of recombinant proteins. To produce higher amounts of recombinant proteins with *P. pastoris*, efficient glycerol and methanol fed-batch procedures could be applied that result in higher cell density and enzyme activity. Shang and co-workers [78] researched for the potential of lignocellulosic biomass on xylanase production by culturing *P. pastoris* in Buffered Complex Glycerol Medium supplemented with corn bran, corncob, cottonseed hull, wheat bran with glycerol–methanol feed. Recombinant xylanase activity was enhanced by 12.2% with 2% (w/v) wheat bran supplementation. Enzymatic hydrolysis of wheat bran can release high-quality proteins, minerals, and phenolic and bioactive carbohydrates which could be advantageous for the *P. pastoris* cell growth and recombinant xylanase expression. The study by Lee [79] evaluated different carbon, i.e., wheat bran, rice bran, the mixture of wheat bran and rice bran (1:1), barley hulls, sucrose, starch, glucose, maltose, glycerol, and nitrogen sources in large scale recombinant xylanase production. Wheat bran supplementation showed the highest effect with an activity value of 1,237 mU.mL^−1^.

Napier grass is a rapidly growing perennial grass that appears similar to sugarcane. Napier grass is easy to cultivate, with yields nearly seven times higher than other grasses. Napier biomass typically comprises 35.0–39.4% cellulose, 19.2–23.4% xylan, and 15.3–19.3% lignin on a dry mass basis [65]. A variety of Napier grasses, i.e., Pakchong 1 (*P*. *purpureum* × *P*. *americanum* L.), Merkeron (*P. purpureum* × *Macroptilium lathyroides* (L.) Urb), Alafal (*P. purpureum* × *P. glaucum* (L.) R. Br.), and sugarcane leaves (*Saccharum officinarum* L.) were investigated for lignocellulosic enzyme production by engineered *P. pastoris* expressing xylanase enzyme gene from *Bacillus firmus* K-1 [80]. The maximum xylanase activity was obtained as 61 U.g^−1^ with sugarcane leaves at 96 h under submerged fermentation, while 50–56 U.g^−1^ enzyme activity was achieved with Alafal, Merkeron, and Pakchong 1 at 72 h. On the other hand, *P. pastoris* presented equivalent cellulase activity of 39.7 and 39.2 U.g^−1^ when utilizing sugarcane leaves or Merkeron as the substrate, respectively.

Maize meal contains 90.9% carbohydrate, while bean pulp having high protein (40–48% w/w) and amino acid (3.6–4.4% w/w) content could be a promising nitrogen source [55, 61, 63]. Maize meal and bean pulp were used to develop recombinant xylanase expression system by *P*. *pastoris* GS115 and Plackett–Burman design and response surface methodology based statistical techniques were employed for medium optimization [81]. 3273 U.mL^−1^ xylanase activity and 0.5794 g.L^−1^ production yield were obtained with the medium including 50 g.L^−1^maize meal and 30 g.L^−1^ bean pulp in shake flask fermentations.

Soybean meal is generated during oil extraction process [82], while rice bran is a by-product of rice milling. Rice bran includes starch, protein, lipid, and dietary fiber in its composition [83]. In a study, medium including crude glycerol (including impurities), soybean hydrolysate (18% w/v total nitrogen) and rice bran (14.63% w/v total nitrogen) as low-cost substrates showed the highest volumetric xylanase activity as 1383.9 U.mL^−1^ [82].

These studies suggest that cereal by-products, e.g., wheat bran, rice bran, corn bran, corncob, maize meal, sugarcane bagasse, bean pulp, soybean hydrolysate and others could be alternative renewable and cheap carbon and nitrogen sources for second generation bio-product formation with *P. pastoris*.

### *P. pastoris* recombinant enzymes in lignocellulosic biomass hydrolysis and conversion

*P. pastoris* has been the most commonly used eukaryotic protein expression host, and a variety of lignocellulose degrading enzymes including hemicellulose, cellulase and laccase have been expressed by *P. pastoris* (extensively reviewed by [45]). Besides being a recombinant protein expression host, *P. pastoris* can be used as whole cell catalyst in the second generation bioprocesses.

Chitosanase is widely used for bioactive chitooligosacchride production. *Streptomyces griseus* HUT 6037 chitosanase gene *CSN5* was expressed by *P. pastoris* GS115 [84]. 90.62 U.mL^−1^ chitosanase activity was obtained in high-density fermentation at 96 h. Recombinant enzyme hydrolysed chitosan and produced a mixture of chitooligosaccharide with 2–4 desirable degrees of polymerization.

Xylooligosaccharides are a promising class of prebiotics capable of selectively stimulating the growth of the beneficial intestinal microbiota against intestinal pathogens. *Thermoascus aurantiacus* GH10 xylanase was expressed by *P. pastoris* and applied to xylan isolated from sugarcane bagasse. A mixture of prebiotic xylooligosaccharides containing mainly xylobiose, xylotriose and xylotetraose were produced [85]. Other than recombinant enzyme, the entire cell might be used as a catalyst repeatedly without activity loss.

A thermostable endo-1,4-β-glucanase GH7 gene from *Aspergillus fumigatus* was expressed by *Pichia pastoris* X-33 [86]. Enzyme performance in biomass degradation for industrial applications was analysed with 1% sugarcane bagasse "in natura", sugarcane exploded bagasse, corncob, rice straw, barley bagasse, or bean straw, supplemented with a commercial cellulase (Celluclast 1.5L, Viscozyme L, Novozyme), and the enzyme showed a high degree of synergy with the commercial coctail in the deconstruction of all the tested lignocellulosic residues, except barley bagasse.

A xylan-degrading gene from *Cellulomonas flavigena* KCTC 9104 was expressed by *Pichia pastoris* X-33 and applied in enzymatic hydrolysis process for sugars production from lignocellulosic biomass [87]. Empty fruit bunch was used a feedstock, and recombinant xylanase showed similar xylose conversion to commercial enzyme.

### Effect of lignocellulosic biomass hydrolysate inhibitors on *P. pastoris*

Prior to microbial fermentation pre-treatment and hydrolysis of lignocellulosic biomass is required. In biomass pretreatment some toxic compounds are released or generated during the breakdown of lignin, hemicellulose deacetylation or pentoses and hexoses dehydration [88, 89]. On the basis of chemical functionality, origin, and impacts on the fermenting microorganisms, toxic by-products of pre-treated lignocellulose can be categorized. The common phenolic compounds of vanillin, syringaldehyde, coniferyl aldehyde, weak acids of aliphatic carboxylic acids; acetic, formic, and levulinic acid, as well as the furan aldehydes of furfural and 5-hydroxymethyl-2-furaldehyde (HMF), which have relatively low toxicity but can be present in high concentrations depending on the pre-treatment conditions and the feedstock, are examples of lignocellulosic hydrolysate inhibitors [90, 91]. For *S. cerevisiae*, the effects of lignocellulose-derived inhibitors on yeast physiology and resistance mechanisms have been thoroughly studied [89, 91–93] and very uncommonly for other yeasts, such as *Pichia stipitis* [94], *Zygosaccharomyces* [95], *Spathaspora passalidarum* [96], *Candida spp.* [97, 98], and others [99]. Depending on the chemical structure of the specific inhibitor and its concentration, inhibitors have various undesired effects causing delayed microbial development, diminished cell viability, and decreased fermentation efficiency. Their primary modes of inhibitory action include cellular membrane damage, redox imbalance, and suppression of crucial enzymes involved in cell metabolism, DNA replication, RNA synthesis, and protein synthesis [100, 101].

The study by Paes et al. [102] investigated the negative effects of lignocellulose derived inhibitors such as acetic acid, furaldehydes (HMF and furfural) and sugarcane hydrolysate on *P. pastoris* physiological and genome-wide transcriptional response. Results revealed that, *P. pastoris* is highly tolerant to lignocellulose-derived inhibitors, especially to acetic acid. *P. pastoris* could able to grow in synthetic media with up to 6 g.L^−1^ acetic acid, 1.75 g.L^−1^ furaldehydes or hydrolysate diluted to 10% (v/v). However, cell metabolism was completely hindered in the presence of 30% (v/v) hydrolysate. Increased concentrations of lignocellulose-derived inhibitors lead to stronger inhibitory effects on yeast metabolism, increasing the time to complete sugar consumption and grow.

Zhou et al. [103] developed an detoxification process of wheat bran hydrolysate to optimize appropriate removal ratio of inhibitors and diminish sugar loss for *P. pastoris* cultivation and xylanase expression under *AOX1* promoter. Optimization of wheat bran hydrolysate was achieved by both individual methods of calcium hydroxide addition, active carbon adsorption, sodium thiosulfate reduction and by the consecutive combination of those steps. In addition, the impact of furfural and 5‑HMF on cell growth and enzyme expression was researched by supplying different concentrations of that inhibitors. *P. pastoris* growth and xylanase B production activities were unaffected below 1.0 g.L^−1^ furfural addition, while higher furfural addition than 1.0 g.L^−1^ suppressed the specific xylanase B synthesis. Increment in 5‑HMF concentration reduced cell growth and enzyme expression. Consequently, 1059.8 U.mL^−1^ xylanase B yield was obtained in detoxified wheat bran hydrolysate which reached 90.9% of that in *Pichia* commercial complex medium [103].

### Sugar industry by-products: molasses and sugarcane bagasse

Molasses, is the major by-product of sugar industry, includes 40–50% (w/w) sugars, i.e., glucose and fructose [125]. Its rich and utilizable sugar composition makes molasses a very attractive renewable carbon source for bio-production of high-demand value-added compounds for variety of markets including detergent, textile, food, and pharma. The potential of molasses as a sole carbon source has been evaluated with various microorganisms. Beet molasses was used in growth medium of *Yarrowia lipolytica* for recombinant laccase [126] and *Dipodascus capitatus* A4C for recombinant lipase production [127]. Zhoukun et al. [108] presented that recombinant α-amylase from *E. coli* could be produced efficiently in *P. pastoris* with an industrial waste of molasses. Çalık et al. [107] evaluated hybrid fed-batch bioreactor operation both with a single and complex carbon source for the production of recombinant human growth hormone. Pre-treated sugar beet molasses hydrolysate including equimolar glucose and fructose was used to provide dual carbon sources in hybrid fed-batch bioprocess and glucose was used as a single carbon source. MH2 is a strategy that was performed in hybrid fed-batch bioreactor operation with molasses, fed-batch periods were 1.5 h with continuous feed stream (*µ* = 0.10 h^−1^) and with 0.5 h batch periods. GH2 is the same strategy applied in this study with glucose as the main carbon source instead of molasses. The maximum recombinant human growth hormone production was 611 mg.dm^−3^ in GH2 at 8 h and 625 mg.dm^−3^ in MH2 at 13.5 h of bioprocess. Recombinant human growth hormone production was increased in MH2 with dual carbon sources, although the productivity is lower due to longer cultivation time.

Sugarcane, cultivated mainly in tropical and sub-tropical countries and the main crop to produce sugar by accounts for nearly 80% global sugar production. Sugarcane bagasse rich in carbohydrates is the main by-product of sugarcane process [128]. Eleven different xylose dehydrogenase (*XDH*) genes from bacterial and fungal origins were identified in silico and six of them cloned to *P. pastoris* successfully. *P. pastoris* expressing the bacterial XDH showed the best acid production reached 37.1 ± 1.9 and 11.7 ± 1.6 g.L^−1^ xylonic acid with the yields of 0.96 ± 0.02 and 0.40 ± 0.06 g xylonic acid/g xylose in mineral medium in bioreactor and sugarcane bagasse hydrolysate in shake–flask, respectively [106].

### High fructose syrup industry by-products

Over the past few years, global high fructose sugar production has increased. China’s annual high fructose sugar production reached 4,150,000 tons in 2020. The general high fructose sugar production procedure from starch follows liquefaction, saccharification, isomerization, and chromatography separation steps [129]. During that consecutive steps, liquid waste including miscellaneous carbohydrates is eluted and the total volume reaches around 415,000 tons per year [130]. The liquid composition of miscellaneous waste carbohydrates (MWC) is complicated, containing a variety of carbohydrates such as glucose, fructose, and oligosaccharides with varying degrees of polymerization, making waste treatment difficult [64]. Gao et. al [105] used miscellaneous waste carbohydrates from high fructose sugars as an alternative carbon source for P_*GAP*_ and P_*AOX1*_ based *P. pastoris* fermentations. The composition of miscellaneous waste carbohydrates contained 802.3 g.L^−1^ total carbohydrates, 480 g.L^−1^ glucose, 92 g.L^−1^ fructose, 103.6 g.L^−1^ maltose, 36.8 g.L^−1^ maltotriose, and 89.9 g.L^−1^ other oligosaccharides varying degrees of polymerization. Fermentation conditions were optimized by evaluating dry cell weight (dcw) and recombinant endo-β-1,3-glucanase activity. In 7 L bioreactor, the highest dcw and enzyme activity was measured, respectively, 69.1 g.L^−1^ and 171.8 U.mL^−1^ in P_*GAP*_ based *P. pastoris* fermentation. The self-designed DO-Stat-Time feeding strategy with P_*AOX1*_ based *P. pastoris* reached 83.6 g.L^−1^ dcw and 212.3 U.mL^−1^ endo-β-1,3-glucanase activity.

### Biodiesel industry by-product: glycerol

Biodiesel, a mixture of different fatty acid methyl esters, is an alternative to fossil fuels. Biodiesel is made from inexpensive sources, such as waste cooking, plant, and animal oils. During biodiesel production, 10% (w/w) crude glycerol which can be also referred as crude glycerine, is generated as the main by-product [32, 131, 132]. In addition to biodiesel industry, soap industry, fatty acid industry and fatty ester industries generate crude glycerol. Crude glycerol is an unavoidable, worldwide highly abundant by-product with low price (~ 0.17$/kg) [133]. The world’s second largest biodiesel producer Brazil reported over 6.7 million cubic meters of biodiesel production as in 2020, which generated about 670,000 m^3^ of crude glycerol (https://www.fas.usda.gov/data/brazil). Biodiesel’s expanding market with 42% annual growth rate has increased the crude glycerol availability and decreased its cost [134]. Valorisation of crude glycerol provides an alternative path both for crude glycerol removal for sustainable environment and as a cheap carbon and energy source for biomass and value-added bioproduct generations [131, 135–137]. However, crude glycerol solutions contain impurities such as methanol, soap, catalysts, salts, non-glycerol organic matter, free fatty acids and water based impurities depending on oil source and trans-esterification process. For instance, the crude glycerol solution from a biodiesel industry in Canada contained 15% (w/w) glycerol, 27% (w/w) soap, 31% (w/v) methanol, and other minor components [138]. Another biodiesel by-product crude glycerol solution consisted of 78% (w/w) glycerol, 1.3% (w/w) methanol, 2.4% (w/w) soap, 2.5% (w/w) water and 0.1% (w/w) NaOH [139]. Since there is a big difference in the composition of crude glycerol solutions, bioprocess parameters should be optimized specifically for each feed, and impurities need to be evaluated in advance considering the final bioproduct quality.

*P. pastoris* can metabolize glycerol efficiently, and at glycerol fed-batch cultivations the cell concentration could reach up to 140 g dcw.L^−1^ [140]. Furthermore, methylotrophic nature of *P. pastoris* provides it with toxic methanol utilization capability. Therefore, crude glycerol including some toxic impurities can be an appealing alternative carbon source for value-added bio-based product generation by *P. pastoris*. A number of research has been conducted on crude glycerol-based *P. pastoris* bioprocess operations. Cui and Ellison, [117] prepared biodiesel waste crude glycerol from a variety of off-the-shelf cooking oils, i.e., canola, corn, sunflower, vegetable and a blend oil of canola and vegetable cooking oil, and used it as a feedstock to produce recombinant spider silk (spidroin) protein. Results showed that *P. pastoris* is highly tolerant to biodiesel by-product glycerol solutions, and irrespective of oil sources, crude glycerol resulted in better cell growth than laboratory-grade glycerol [117].

Singsun et al., [115] collected waste glycerol from two different companies, i.e., E-ester company, and Meacham pork cracker community enterprise and investigated the influence of treatment and different concentrations of glycerol solution on *P. pastoris* cell growth. 1%, 2%, and 5% (w/v) crude glycerol containing media were used for *P. pastoris* cultivation and the maximum cell concentration was attained with 5% untreated waste glycerol [115]. In the study by Luo, et al. [113], the effect of crude glycerol impurities on *P. pastoris* cell growth and recombinant β-mannanase expression was investigated and results showed that salts and methanol did not show toxicity, while 0.2% and 0.3% (w/v) soap inhibited the fermentation. Under desirable conditions, untreated 5% crude glycerol usage in bioreactor level resulted in 77.9 g.L^−1^ biomass and 10,900 U.mL^−1^ β-mannanase activity that is higher than that of obtained from pure glycerol [113]. In another study, biodiesel industry by-product crude glycerol containing 60% (w/w) glycerol, < 1% (v/v) methanol, 20.6% (w/w) methyl ester and grease, and 14.2% metal saponification having 0.53% (w/w) Fe^3+^, 9.65% (w/w) Na^+^, 1.37% (w/w) K^+^, and 0.05% (w/w) Ca^2+^ was examined for the effects of these impurities on *P. pastoris* cell growth, recombinant lipase enzyme expression and lipase activity [111]. Response surface methodology was applied to quantify the cumulative effects of these contaminants. The study showed that cells reach the stationary phase earlier due to impurities, adding 1.1–6.8% crude glycerol improves the cell growth, while the higher concentrations inhibits the growth. In addition, 15,977 U.mg^−1^ lipase activity that is higher than that of pure glycerol was obtained and the key components responsible for the enhanced activity were specified as Na^+^, Ca^2+^, and grease in crude glycerol [111]. Likewise, crude glycerol was used to produce recombinant lectin, and the maximum yield was attained as 265 ± 13 mg.L^−1^ through optimization of bioreactor operation parameters, e.g., pH, aeration, agitation, and temperature [110]. Eleven different glycerol samples were obtained with methanolysis of soybean oil using different catalysts and purification steps, and they were used for the production of recombinant α-amylase [116]. Crude glycerol prepared with potassium or sodium hydroxide provided 1.5–2 times higher cell densities than those of commercial pure glycerol, and 3.5 U.mL^−1^ enzyme activity was achieved. *Candida antarctica* lipase B gene was expressed in *P. pastoris* under the constitutive P_*GK1*_ promoter using crude glycerol as a carbon source [114] and the optimal conditions were determined as starting the batch phase with 100 g/L glycerol containing minimal medium and then four subsequent pulses of 25 g.L^−1^ crude glycerol, that reached 50,041 U.L^−1^ lipase B activity. A fungal laccase production process was designed by recycling the *P. pastoris* biomass and replacing pure glycerol with crude glycerol. 2,343 U.L^−1^ laccase was produced after 48 h. Recycling of free cells for 6 times produced > 8800 U laccase, while recycling of immobilized cells for 18 batches resulted with 27,681–33,926 U laccase production with pure/crude glycerol media [109].

Sago fiber, which contains 50–60% residual starch and other lignocellulosic materials, is one of the potential agricultural wastes in Malaysia [141]. Sago bioethanol liquid waste (SBLW) is generated following the bioethanol fermentation by *S. cerevisiae* using sago fiber hydrolysate. Wahida et al. [112] used sago bioethanol liquid waste (SBLW) to produce recombinant laccase by *P. pastoris* GS115. The main component of SBWL was found to be glycerol (3.25 g.L^−1^), followed by glucose (0.41 g.L^−1^), lactic acid (0.18 g.L^−1^), and ethanol (0.18 g.L^−1^). In comparison with buffered complex methanol medium, supplementation of 40% (v/v) sago bioethanol liquid waste with 1.0% (w/v) yeast extract resulted in 1.2-fold and 1.5-fold increased biomass concentration and laccase titer, respectively [112].

### Different strategies for valorisation of wastes and by-products with *P. pastoris*

#### Co-culture with other microorganisms

Co-culture strategy benefits from conversion with sequential or simultaneous fermentation of more than one microorganism for valorisation of wastes into value-added products. Kitchen wastes come out in large quantities (100 million tons annually) and their traditional disposal methods such as incineration and landfill lead to environmental pollution. Kitchen waste constitutes of 47.3 ± 0.6% starch, 25.0 ± 0.8% lipid and 6.8 ± 0.4% protein on dry weight basis. For kitchen waste pre-treatment, *P. pastoris* strains were designed for lipase, amylase and glucosidase expression and, respectively, 90 U.mL^−1^, 385.4 U.mL^−1^ and 247.3 U.mL^−1^ enzyme activities were achieved [142]. Three engineered *P. pastoris* strains for secretion of hydrolytic enzymes were co-cultured with *Bacillus amyloliquefaciens* HM618 that is engineered to produce fengycin, and 6.6 times higher fengycin concentration was obtained from kitchen wastes compared to the production with pure culture [142]*.*

Two yeast co-culture strategy was also applied by Zhang et al. [37] to produce ethanol directly from wheat straw benefiting from recombinant enzyme expression for simultaneous saccharification and fermentation. Wheat straw is lignocellulosic biomass consisting of cellulose and hemicellulose that requires enzymatic pre-treatment to release fermentable sugars; glucose and xylose. Endoglucanase (EC 3.2.1.4), cellobiohydrolase (EC 3.2.1.91 or exoglucanase) and β-glucosidase (EC 3.2.1.21) are types of cellulases that are responsible for saccharification, while xylanases: endoxylanase (endo-1, 4-β-D-xylanase, EC 3.2.1.8) and β-xylosidase (EC 3.2.1.37) catabolise xylan to xylose. Cellulose-utilizing engineered *S. cerevisiae* BY47434A co-expressing three cellulase enzymes and xylan-utilizing engineered *P. pastoris* GS115 co-expressing endo-1,4-β-D-xylanase and β-xylosidase enzymes were cultured with wheat straw to provide simultaneous saccharification and fermentation. 32.6 g.L^−1^ ethanol from 100 g.L^−1^ total sugar produced with co-culture strategy at 70 h of fermentation using wheat straw. Co-culture strategies are promising second generation bioprocess operations to decrease cost and enhance efficiency.

#### Metabolic engineering of *P. pastoris*

Recombinant protein production creates metabolic burden as it takes precursors from the central carbon metabolism, consumes redox and energy co-factors, and can lead to energetic imbalances in the metabolism. These cellular mechanisms are diverted from their evolutionary objective of cell development and maintenance. Adequate cellular capacities for translation, folding, posttranslational modifications, and localization of the protein are necessary for the high-yield recombinant protein synthesis. As strong recombinant production processes are highly demanding, some molecular functions and metabolites may be limited and result in bottlenecks [143–145]. Metabolic engineering have been used to solve the limitations of protein expression by boosting the availability of precursors, maintaining cellular redox balance, and enhancing energy efficiency. In addition to aforementioned reasons, for sustainable bioeconomy genetic manipulations on metabolic pathways are required to evolve the strain of interest to utilize substrates that cannot be consumed naturally by microorganisms.

**Fig. 2:**
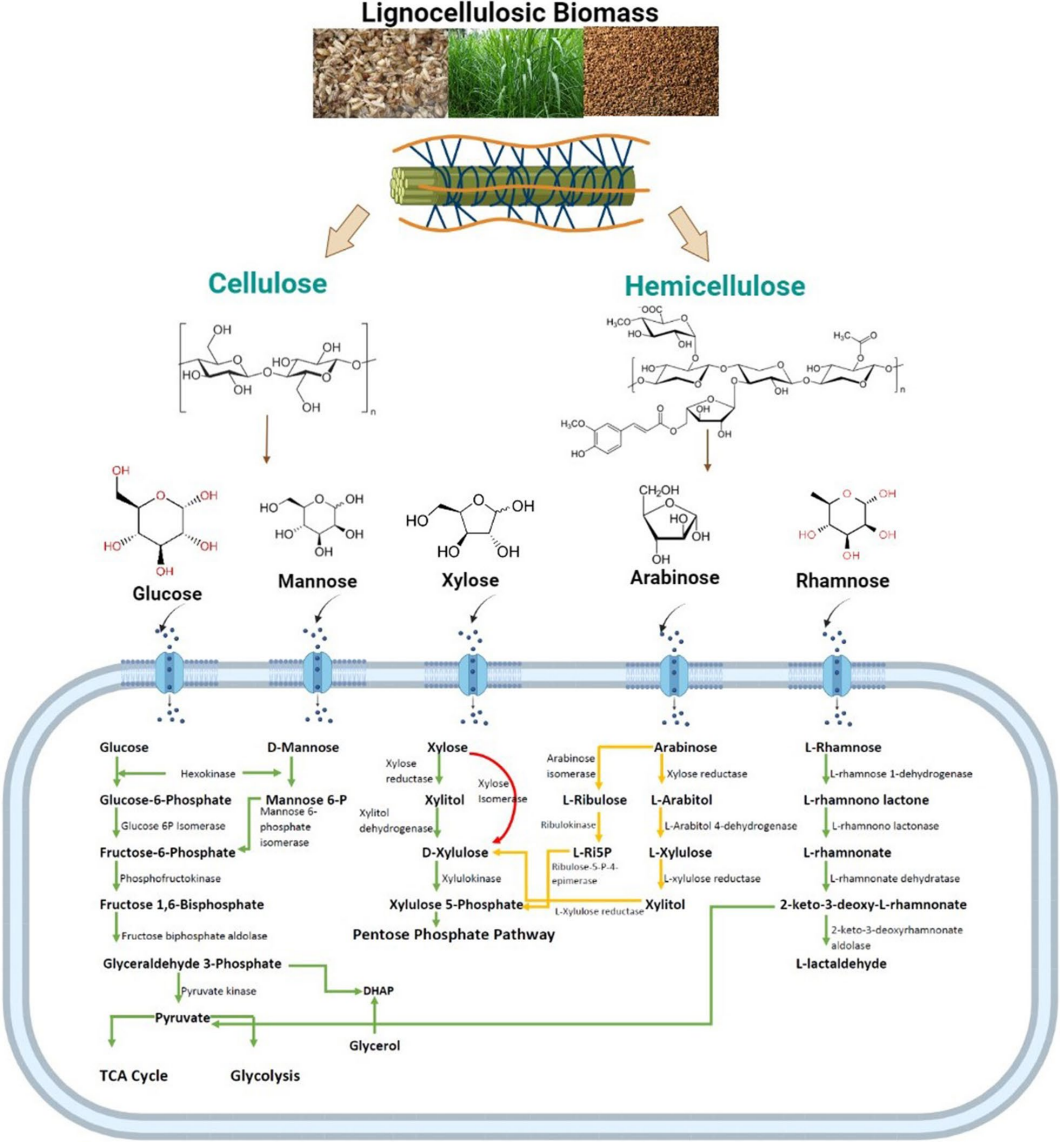
Hexose and pentose metabolism of the yeast cell. Green arrows indicate naturally occurring metabolic pathways in *P. pastoris*, Red arrows indicate integrated heterologous metabolic pathways. Yellow arrows indicate potential metabolic engineering targets for *P. pastoris* that have not been constructed, yet. L-Ri5P: L-ribulose 5-Phosphate, DHAP: dihydroxyacetone phosphate, TCA cycle: tricarboxylic acid cycle

The oxidoreductase route is extensively used in xylose metabolism, in which D-xylose is transformed to xylitol by xylose reductase and then to xylulose by xylitol dehydrogenase. A xylulokinase converts xylulose to xylulose-5-phosphate, which then enters the pentose phosphate pathway (Fig. 2). In prokaryotes, there is an alternate mechanism that converts D-xylose directly to xylulose using a xylose isomerase [51]. Heterologous protein expression associated with xylose catabolism was first achieved with metabolic engineering studies by Li et al. [51]. A heterologous XI pathway was integrated into *P. pastoris* genome, as well as an evolutionary engineering technique was employed. In another study, as high as 80% (w/w) conversion of D-xylose whether in pure form or as a crude hemicellulose hydrolysate was achieved in *P. pastoris* by inserting the genes of xylose reductase from *P. stipitis* and glucose dehydrogenase from *B. subtilis* [121]*.*

Another *P. pastoris* metabolic engineering study targeted direct isobutanol production from sugarcane trash hydrolysate [119]. *P. pastoris* was engineered for heterologous xylose isomerase and endogenous xylulokinase overexpression to consume both C5 and C6 sugars in biomass. For isobutanol production, endogenous amino acid biosynthetic pathway and 2-keto acid degradation pathway were overexpressed. Resulting strain produced isobutanol at a titer of 48.2 ± 1.7 mg.L^−1^ directly from minimal medium containing sugarcane trash hydrolysates as the sole carbon source [119].

For utilization of lignocellulosic biomass, *P. pastoris* was engineered for constitutive co-expression of *Aspergillus niger* β-glucosidase (*An*BGL1) and endoglucanase (*An*EG-A), and *Trichoderma reesei* exoglucanase (*Tr*CBH2). This engineered strain was able to grow on cellobiose and amorphous carboxymethylated cellulose [123]. In another study, lignocellulosic biomass utilizing *P. pastoris* strain was developed using an assembly of enzyme complexes. The chimeric endoglucanase cCelE from *Clostridium thermocellum* and the xylanase XynB from *Clostridium cellulovorans* were chosen as the enzyme subunits and attached to a recombinant scaffolding protein mini-CbpA from *Clostridium cellulovorans* to form the enzyme complexes. These complexes effectively decomposed carboxymethylcellulose and xylan [124].

Another alternative carbon source is acetate that can be obtained in a variety of ways, including hydrolysis or pyrolysis of cellulosic biomass, chemical or microbial catalysis, and anaerobic fermentation in treated wastewater. Metabolic engineering efforts was also applied to increase acetate tolerance of *P. pastoris* [122]. From a kinase-deficient *P. pastoris* library, a native (serine/threonine–protein kinase) HRK1 kinase that plays an essential role in acetate tolerance was discovered. Co-overexpression of HRK1 and the acetyl-CoA synthesizing genes increased acetyl-CoA-dependent 6-methylsalicylic acid production and enhanced acetate tolerance [122].

From the seven currently known carbon dioxide fixation pathways found in nature, the Calvin–Benson–Bassham (CBB) cycle is seen as the main driver of primary production on Earth [146–149]. Ribulose-1,5-biphosphate carboxylase-oxygenase (RuBisCO) is one of the most abundant enzymes found in the biosphere and the key enzyme of cyclic pathway that converts about 90% of inorganic carbon into biomass.

The xylulose monophosphate (XuMP) cycle for methanol assimilation of *P. pastoris* occurs in peroxisome and this pathway shares high similarities with the CBB cycle. Like transferring C1 molecule to a sugar phosphate forming a C–C linkage. Alcohol oxidase enzymes AOX1 and AOX2 oxidize methanol to formaldehyde, and by dihydroxyacetone synthase (DAS1 and DAS2) formaldehyde further react with xylulose 5-phosphate (Xu5P) and dihydroxyacetone (DHA) and glyceraldehyde-3-phosphate (GAP) are formed. As a result, from three methanol molecule, one molecule of GAP in which utilized for biomass generation, is produced. Similarly, in carboxylation reaction, autotrophic organisms add CO_2_ to ribulose-1,5-bisphosphate (RuBP) to generate 3-phosphoglycerate (3-PGA) catalysed by RuBisCO. The reaction follows the phosphorylation and reduction of 3-PGA to GAP. Thus, by addition of eight heterologous genes and deletion of three native genes, the XuMP cycle of *P. pastoris* was resembled to a synthetic CBB cycle and *P. pastoris* was converted from heterotroph to autotroph. The resulting strain can continuously grow on CO_2_ as a sole carbon source at a µ_max_ of 0.008 h^−1^ and by adaptive laboratory evolution (ALE) the specific growth rate was further improved to 0.018 h^−1^ [120]. Single nucleotide polymorphisms (SNPs) occurring in the genes encoding for phosphoribulokinase and nicotinic acid mononucleotide adenylyltransferase were found infleuntial on the improved autotrophic phenotypes after ALE. The reverse engineered SNPs resulted in lower enzyme activities in putative branching point reactions and in reactions involved in energy balancing [150].

## Conclusion and future perspectives

Without doubt, with the ever-growing bioproduct and biorefinery markets, the yeast expression systems will continue to thrive. This article points out the necessity of efficient second generation *P. pastoris* strain and bioprocess designs for cost-effective and sustainable fermentation processes to obtain tailor-made bioproducts from renewable alternative feedstocks. Ever-growing population create high demand for commodity products and cause unsustainable model of production and consumption based on increased use and depletion of resources. To establish a robust bioeconomy, economically viable conversion of low-cost renewable feedstock into bioproducts and biofuels is of utmost importance. Non-conventional yeast *P. pastoris* has been receiving more attention due to its alternative metabolic routes to produce tailor-made metabolites, utilisation capability of a variety of complex substrates, and well-established industrial bioprocess operations. Second generation *P. pastoris* platforms define sustainable biomass and bioproduct generations by valorisation of wastes and by-products. Recycling of by-products and wastes contributes to eco-friendly and sustainable biotechnological production.

The most commonly used low-cost second-generation substrate for *P. pastoris* bioprocess operation has been glycerol. *P. pastoris* can reach hundreds of grams per litre cell densities using glycerol. For the design of gene expression cassettes there is a number of constitutive and glycerol regulated promoters [2, 6]. Biodiesel’s expanding market with 42% annual growth rate makes crude glycerol an unavoidable and highly abundant by-product [134]. Recycling of crude glycerol is very important for sustainable environment, and furthermore, it provides a cheap carbon and energy source for biomass and value-added metabolite production [131, 135–137]. Furthermore, *P. pastoris* high tolerance against toxic contaminants of crude glycerol such as methanol and salts [113] makes it as a promising alternative feedstock. Higher biomass and recombinant protein yields were also achieved with crude glycerol compared to pure glycerol due to stimulating effects of Na^+^, Ca^2+^, and grease presence [111, 113, 114, 117].

Lignocellulose is the most abundant biomass on Earth with an annual global production of about 181.5 billion tonnes [43], about 4.6 billion tonnes of lignocellulosic biomass residues are produced as agricultural residues, of which only about 25% are used intensively [44]. Million metric tonnes of agri-food wastes and billion tonnes of household food and beverage wastes have been generated each year (Table 1). In addition, these wastes are rich in carbon and their untreated disposal leads to environmental pollution and health problems. Alternative to commercial fermentation media components; industrial by-products and agri-food wastes could be used as carbon, nitrogen, or other essential nutrient sources in the development of second generation bioprocess operations. Metabolic pathway engineering and adaptive evolution strategies enabled *P. pastoris* strains to utilize lignocellulosic biomass components; however, there is still room for improvement. Consolidated cellulose and hemicellulose degradation pathways need to be constructed within the *P. pastoris* to enhance degradation efficiency of agri-food wastes and increase the availability of monosaccharides for one-pot bioprocess designs. Xylose and arabinose are the major constituents of hemicellulose, large quantities of which are found in agricultural wastes. Although, xylose utilization capabilities of *P. pastoris* strains have been investigated to some extent, arabinose did not take any attention, yet. Arabinose assimilation pathway could be constructed by integration of three heterologous enzyme genes (Fig. 2) to provide precursors for cell growth and bioproduct formation. In addition, xylitol production from arabinose could be achieved by integration of metabolic pathway employing only three different enzymes (Fig. 2). In the years to come, *P. pastoris* metabolic engineering and adaptive laboratory evolution studies will possibly target both utilisation of different lignocellulosic biomass constituents such as arabinose and improving the endogenous assimilation pathways for second generation bioprocesses. In addition, *P. pastoris* high tolerance to lignocellulose derived inhibitors will widen the usage of lignocellulosic feedstock. Co-culture strategy benefits from conversion with sequential or simultaneous fermentation of more than one microorganism is also a recently emerging tool. Metabolically engineered *P. pastoris* strains show great promise in co-culture studies for direct conversion of wastes into value-added products.

In spite of the technological and scientific advancements on *P. pastoris*, second generation *P. pastoris* cell factory designs and applications on cheap sustainable raw materials are still scarce to fulfil sustainability requirements for circular bioeconomy. In the years to come, *P. pastoris* strain engineering will expected to be focused on the creation of more sustainable yeast strains to boost the transition to a more sustainable industry based on renewable raw materials that can utilize low-cost raw materials and agri-food waste streams for production of large volume commodity products. Modern bioprocess engineering and advances in omics technology*, *i.e., genomics, transcriptomics, proteomics, secretomics, and interactomics, will allow the design of novel genetic circuits and metabolic pathways to develop second generation *P. pastoris* strains and bioprocess operations. The successful replacement of traditional carbon sources by agri-food industry by-products will greatly decrease the cost of large-scale fermentations, further relieve the high burden waste treatment, and establish the second generation cell engineering and production procedures for industries employing *P. pastoris* and other microorganisms.

## Data Availability

Not applicable.

## Abbreviations

SCP: Single cell protein
EMA: European medicine agency
EFSA: European food safety authority
FDA: USA Food and Drug Association
QPS: Qualified presumption of safety
P_*AOX1*_: Alcohol oxidase 1 promotor
P_*GAP*_: Glyceraldehyde 3-phosphate dehydrogenase promotor
P_*MOX*_: Methanol oxidase promotor
HRK1: Serine/threonine protein kinase
MWC: Miscellaneous waste carbohydrates
dcw: Dry cell weight
